# An Efficient Outlier Detection with Deep Learning-Based Financial Crisis Prediction Model in Big Data Environment

**DOI:** 10.1155/2022/4948947

**Published:** 2022-08-16

**Authors:** Yalla Venkateswarlu, K. Baskar, Anupong Wongchai, Venkatesh Gauri Shankar, Christian Paolo Martel Carranza, José Luis Arias Gonzáles, A. R. Murali Dharan

**Affiliations:** ^1^Department of Computer Science and Engineering, BVC College of Engineering, Rajahmundry, East Godavari District, Andhra Pradesh, India; ^2^Kongunadu College of Engineering and Technology, Thottiam, Tamilnadu, India; ^3^Department of Agricultural Economy and Development, Faculty of Agriculture, Chiang Mai University, Chiang Mai, Thailand; ^4^Manipal University Jaipur, Jaipur, Rajasthan, India; ^5^Universidad de Huánuco, Huánuco, Peru; ^6^Pontificia Universidad Católica Del Peru, San Miguel, Peru; ^7^Debre Berhan University, Debre Berhan, Ethiopia

## Abstract

As Big Data, Internet of Things (IoT), cloud computing (CC), and other ideas and technologies are combined for social interactions. Big data technologies improve the treatment of financial data for businesses. At present, an effective tool can be used to forecast the financial failures and crises of small and medium-sized enterprises. Financial crisis prediction (FCP) plays a major role in the country's economic phenomenon. Accurate forecasting of the number and probability of failure is an indication of the development and strength of national economies. Normally, distinct approaches are planned for an effective FCP. Conversely, classifier efficiency and predictive accuracy and data legality could not be optimal for practical application. In this view, this study develops an oppositional ant lion optimizer-based feature selection with a machine learning-enabled classification (OALOFS-MLC) model for FCP in a big data environment. For big data management in the financial sector, the Hadoop MapReduce tool is used. In addition, the presented OALOFS-MLC model designs a new OALOFS algorithm to choose an optimal subset of features which helps to achieve improved classification results. In addition, the deep random vector functional links network (DRVFLN) model is used to perform the grading process. Experimental validation of the OALOFS-MLC approach was conducted using a baseline dataset and the results demonstrated the supremacy of the OALOFS-MLC algorithm over recent approaches.

## 1. Introduction

With the dynamic expansion of the financial marketplace, enterprises might increase lower-cost deposit from the financial marketplace to quicken their improvement, and investor uses the process of the financial marketplace to finance and acquire high revenues [1]. But current companies are confronting progressively unsuccessful marketplace environments, and risk continuously provides operators a problem. The features of the current enterprise environment are mostly replicated in the rapid development of information technology, economic globalization, changes in business models, management methods, and customer orientation. This factor is influenced by technology, society, economy, and politics. The existing procedure of current companies is a method where different types of risks are endlessly produced and solved [2]. Over the last few years, the most important constraint for positioning resourceful devices is extremely pertinent to small and medium-sized enterprises (SMEs) for predicting economical faults and business loss. SMEs need business management for observing the modus operandi and inspect whether it is relevant to attain the determined objectives [3]. This model is portrayed through a series of firm rules, and some approaches are called “controls,” which guarantee the structure of the enterprise organization. At last, the requirement is stimulated for intermittent assessments [4]. As a result, detecting and estimating the development of corporate entitie make it easy to understand by the high dynamism that proposes to be a complicated process. It is important to progress that served as the inspection of efficiency in the economy [5].

Over the last few years, with the advance of the economic crisis of businesses all over the world, the enterprises are paying more interest in the field of FCP [6]. For a financial or company organization, it is vital to model earlier and reliable predictive models for forecasting the possible risk of the business status of economic failure. FCP usually yields a dual classification model that was resolved rationally [7, 8]. The outcome from the classification models is classified as a failure and nonfailure status of enterprise [9]. Previously, several classification methods are designed with different areas of concern for FCP. Usually, the proposed predictive method classified into Artificial Intelligence (AI) or statistical methods [10].

El-Kenawy et al. [11] present a modified binary grey wolf optimization (MbGWO) dependent upon stochastic fractal search (SFS) for identifying essential features with attaining the exploration and exploitation balances. Next, the diffusion procedure SFS implemented an optimum solution of modified GWO by utilizing the Gaussian distribution approach to arbitrary walk from a development procedure. Sankhwar et al. [12] establish a new predicting structure for the FCP method by the hybrid IGWO and fuzzy neural classifier (FNC). The proposed IGWO-based FS approach was utilized for discovering an optimum feature in the financial data. To classifier drives, FNC was utilized.

The authors in [13] present Bolasso (Bootstrap-Lasso) that chooses consistent and relevant features in a pool of features. The consistent feature selection (FS) was determined as the robustness of selecting features in terms of alterations in the dataset Bolasso created shortlisted feature is then executed for several classifier techniques such as K-NN, SVM, RF, and NB for testing their prediction accuracy. Kim et al. [14] present globally optimizing SVM, signified by GOSVM, a new hybrid SVM approach structured for optimizing FS, sample selection, and kernel parameters. This study presents GA for concurrently optimizing several heterogeneous designed factors of SVMs. Ghosh et al. [15] present a wrapper-filter group of ACO, whereas it can be established subset estimation utilizing a filter approach before utilizing a wrapper approach for reducing computational complexity. A memory for keeping optimum ants and feature dimensional-dependent pheromone upgrade has also been utilized for executed FS from a multiobjective approach. This presented method is estimated on several real-life datasets, obtained in the UCI-ML repository and NIPS2003 FS challenge, utilizing KNN and MLP techniques.

This study develops an oppositional ant lion optimizer-based feature selection with a machine learning-enabled classification (OALOFS-MLC) model for FCP in a big data environment.

- To handle the big data in the financial sector, the Hadoop MapReduce tool is employed
- The proposed OALOFS-MLC model designs a novel OALOFS technique to choose an optimal subset of features which helps in attaining improved classification results
- The deep random vector functional links network (DRVFLN) model is exploited to perform the classification process
- The experimental validation of the OALOFS-MLC algorithm was performed using a benchmark dataset

The remaining section in this paper as follows: Section 2 describes the proposed model, and Section 3 describes the results and discussions.  Section 4 concludes the paper.

## 2. The Proposed Model

**Figure 1:**
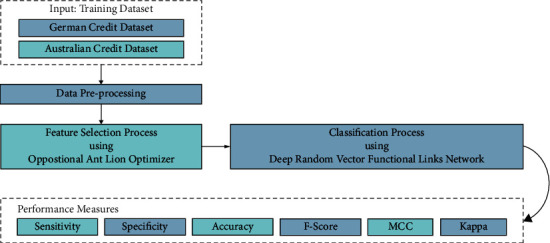
Block diagram of OALOFS-MLC approach.

In this study, a novel OALOFS-MLC model was established for FCP in a big data environment. Besides, the presented OALOFS-MLC model designs a novel OALOFS technique to choose an optimal subset of features which helps in attaining improved classification results. Furthermore, the DRVFLN model exploited to perform the classification process.  Figure 1 depicts the block diagram of the OALOFS-MLC approach.

### 2.1. Hadoop MapReduce

Hadoop is a group of tools and technologies with considerable improvement; the application in the Hadoop technology solution is moderately outstanding in the public sources [16]. Map Reduce is the building block of Hadoop. It can be a corresponding program design framework. Map Reduce utilized for solving the problems of similar operations and analysis in largescale datasets. The foundation of the term Map Reduce is defined by the two fundamental procedures: the mapping process Map and the inductive process Reduce. Map Reduce implements the process simultaneously on a sequence of working nodes. Every node makes use of similar coding for processing the succeeded information without data communication. Map Reduce makes developers no longer assume the fundamental information while designing largescale dataset processing applications, understanding the consistent interface according to the operation that considerably decreases the improvement complexity and progresses the enlargement effectiveness.

### 2.2. Design of OALOFS Technique

In this study, the presented OALOFS-MLC model designs a novel OALOFS technique to choose an optimal subset of features which helps in attaining improved classification results. Reference [17] proposed an Ant Lion optimizer (ALO) that is a nature-inspired metaheuristic approach that simulates the hunting system of antlion in catching their prey. Constructing traps, random walking (RW) of ants, catching ants, reconstructing, and traps entrapment of ants in traps are different measures of the ALO. The antlion is generally known as doodlebugs. Larvae and adults are 2 metamorphic phases in their life cycle. ALO is stimulated by the hunting system characteristics of antlions. The steps included in the calculation of the parameter of the solar cell due to the impact of the environmental condition are given below:

**Step 1 .**

Initialization:

An initialized population of ants is represented as *X*_Ant_=(*x*_1_, *x*_2_,…, *x*_*N*_) and antlion is referred to *X*_as *X* Antlion_=(*x*_1_, *x*_2_,…, *x*_*N*_) produced within the searching region of the parameter as *x*_Antlion_=*I*_*pv*_, E_g_, *μ*, *R*_*s*_, *β*_*s*_, *R*_*p*_, *β*_*p*_, *n*, *α* for ant and (*x*_Antlion_=*I*_*pv*_, E_g_, *I*_0_, *μ*, *R*_*s*_, *β*_*s*_, *R*_*p*_, *β*_*p*_, *n*, *α*) correspondingly, where the size of the population can be represented as *N*. The searching region of the parameter for Photowatt PWP201 PV module and R.T.C. evaluate the present value of each ant and antlion and describe the fitness values, discover the optimal antlions and it is represented as elite. Fix the maximal amount of iterations as max_iter.

**Step 2 .**

Constructing the trap:

For all the ants, antlion is preferred by Roulette wheel selection according to the optimal fitness of antlion for constructing the trap for ant [18].

**Step 3 .**

RW of ant:

The ant moves randomly to search for food and it can be arithmetically formulated in the following equation:

(1) $$\begin{array}{c} X\left(it\right)=\left[O\text{,cum sum}\left(2r\left({\text{it}}_{1}\right)-1\right),\ldots ,\text{cum sum}\left(2r\left({\text{if}}_{{\text{mox}}_{-}\text{iter}}\right)-1\right)\right]. \end{array}$$

In (1), cum sum can be represented as a cumulative sum. *n* refers to the maximal amount of iterations. it indicates the step of RW and *r*(it) denotes a stochastic function as follows:

(2) $$\begin{array}{c} r\left(\text{it}\right)\left\{\begin{array}{cc} 1, & \text{if random.random}>0.5,\\ 0, & \text{if random.random}\leq 0.5. \end{array}\right) \end{array}$$

In (2), random. random () is a randomly produced integer that lies within the range of [0,1]. The normalization formula of the RW of ant from (1) is utilized for maintaining the location of the ant in the searching region.

(3) $$\begin{array}{c} X^{\left(\text{it}\right)}=\frac{\left(X^{\left(\text{it}\right)}-a^{\left(\text{it}\right)}\right)+\left(d-c\right)}{\left(b^{\left(\text{it}\right)}-a^{\text{it}}\right)}+c. \end{array}$$

In (3), *a*, *b*, *c*, and *d* are minimal of RW and are maximal of RW, and lower and upper bounds of the parameter correspondingly. An RW can be normalized for every parameter. For RW of ant (*R*_*A*_), antlion is designated by the Roulette wheel and for elite antlion (*R*_*E*_), and they are normalized and implemented.

**Step 4 .**

Trapping of ants:

The mathematical expression of trapping ants can be given in the following equation:

(4) $$\begin{array}{c} c_{i}^{\left(\text{it}\right)}=x_{{\text{Antlion}}_{j}}+c^{\left(\text{it}\right)},\\ d_{i}^{\left(\text{it}\right)}=\chi_{{\text{Antlion}}_{j}}+d^{\left(\text{it}\right)}. \end{array}$$

In Equation (4), *i* and *j* indicate the indices of designated ant and antlion correspondingly.

**Step 5 .**

Sliding of ant toward antlion: the antlion throws and at the edge of the trap for sliding the ant toward the trap once an ant tries for escaping. It is formulated by

(5) $$\begin{array}{c} d^{\left(\text{it}\right)}\frac{d^{\left(\text{if}\right)}}{I},\\ c^{\left(\text{it}\right)}=\frac{c^{\left(\text{it}\right)}}{I}, \end{array}$$

where *I*=10^*w*^*ir*/mox_−_iter., *i* indicates the present iteration and max_−_iter denotes the maximal amount of iterations. *w* indicates a constant that relies on the iteration as follows:

(6) $$\begin{array}{c} \text{w}=\left\{\begin{array}{c} 2\text{when it}>0.1\;{\text{max}}_{-}\text{iter,}\\ 3\text{when it}>0.5\;{\text{max}}_{-}\text{iter,}\\ 4\text{when it}>0.75\;{\text{max}}_{-}\text{iter,}\\ 5\text{when it}>0.9\;{\text{max}}_{-}\text{iter,}\\ 6\text{when it}>0.95\;{\text{max}}_{-}\text{iter}. \end{array}\right) \end{array}$$

**Step 6 .**

Catching prey and *re* ‐constructing pit:

The fitness of the novel location of the ant was estimated. When the ant becomes fitter (viz., location of the antlion) when compared to the respective antlion, the ant has been trapped by the antlion and the antlion reconstructs the trap for the following hunt.

(7) $$\begin{array}{c} \chi_{AL_{j}}^{t}=x_{A_{i}}^{r}\text{if}\left|RMSE\right|\left(x_{A_{i}}^{t}\right)>\left|RMSE\right|\left(x_{AL_{j}}^{t}\right). \end{array}$$

In (7), *x*_*AL*_ represents the location of the antlion. This procedure is considered as catching prey and reconstructing the pit at the location where there is a higher probability of catching ant for the following iterations.

**Step 7 .**

Elitism:

It can be the procedure for maintaining the location of optimal antlion (elite) by optimized technique. It can be performed by the following equation:

(8) $$\begin{array}{c} \chi_{A}^{t}=\frac{R_{A}^{t}+R_{E}^{t}}{2}. \end{array}$$

In (8), *t* denotes the present iteration and *x*_*a*_^*t*^ indicates the location of ant.

**Step 8 .**

Upgrade elite when an antlion becomes fitter when compared to elite.

**Step 9 .**

End when the stopping condition is accomplished otherwise return to Step 3 to start the following iteration.

For improving the efficiency and performance of ALO, the study presents a revised edition of the technique using the concept of opposition-based learning (OBL). From the abovementioned statement, ALO, as a member of a population‐based optimization algorithm, initiates a set of primary solutions and tries to increase the efficiency toward the optimal solution. During the nonexistence of prior knowledge regarding the solution, the randomly initialized technique is applied for generating a candidate solution (rat first position). The convergence speed and performance are strongly associated with the distance of the first solution from the finest solution. In another word, the process has improved performance when the arbitrarily created solution has the lowest value when compared to the objective function. Based on the concept and to increase the chance of finding the global optima and the convergence speed of typical ALO, this study presents a revised edition of the approach named OALO. In the OALO, the initial iteration of the process afterward produces the first arbitrary solution, and the opposite position of every solution would be produced according to the conception of the opposite number. To determine the new initialized population, it is essential to describe the conception of the opposite number. Given that *n* ‐dimension vector *X* is defined by the following equation:

(9) $$\begin{array}{c} X=\left(x_{1},x_{2},\ldots ,x_{N}\right). \end{array}$$

In equation (9), *x*_*i*_ ∈ [*x*_*i* min_, *x*_*i* max_]. Then, the opposite point of *x*_*i*_, that is represented as $\bar{x_{i}}$, in the following:

(10) $$\begin{array}{c} \bar{x_{i}}=\left(x_{i\text{ max}}+x_{i\text{ min}}\right)-x_{i}\;i=1,2,\ldots ,N. \end{array}$$

To employ the concept of opposite number in the initialized population of OALO, assume *x*_*i*_ as an arbitrarily produced solution in *N*-dimension problem space (that is, solution candidate). For that arbitrary solution, its opposite would be produced by (10) and represented as $\bar{x_{i}}$. Next, these two solutions $\left(i.e.,x_{j}\text{and}\bar{x_{i}}\right)$ are estimated by the objective function *f*. Hence, when $f\left(\bar{x_{i}}\right)$ is superior to $\left(x_{i}\right)\left(\text{viz}.,f\left(\bar{x_{i}}\right)<f\left(x_{i}\right)\right)$, then the agent (*x*_*i*_) would be substituted with $\bar{x_{i}}$; or else, continued with *x*_*i*_.

The fitness function (FF) of the OALO approach assumes the classifier accuracy and the count of chosen features. It maximizes the classifier accuracy and minimizes the set size of chosen features. Thus, the subsequent-FF has been utilized for evaluating individual solutions, depicted as follows:

(11) $$\begin{array}{c} \text{Fitness}=\alpha *\text{ErrorRate}+\left(1-\alpha \right)*\left(\frac{\text{Number of Selected Features}}{\text{Total number of features}}\right), \end{array}$$

whereas ErrorRate signifies the classifier error rate utilizing the chosen features. *α* is utilized for controlling the significance of classifier quality and subset length. During the experiments, *α* is fixed to 0.9.

### 2.3. Data Classification Process

**Figure 2:**
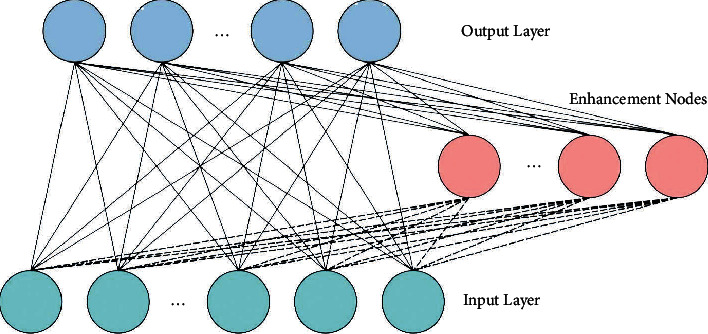
Structure of RVFLN.

Finally, the DRVFLN model is exploited to perform the classification process. The DRVFLN network is wide of shallow RVFL networks assuming deep or representation learning. An input to every layer from the stack result of the prior layer whereas every layer constructs an internal representation of input data [19]. At this point, regarding a stack of *L* hidden layers (HL), they all have the same count of hidden nodes *N*. In order to ease representations, neglect the bias term in the equation.  Figure 2 depicts the framework of the RVFLN technique.

Afterward, the resultant primary HL is defined as follows:

(12) $$\begin{array}{c} H^{\left(1\right)}=g\left(XW^{\left(1\right)}\right). \end{array}$$

All the layers *l* > 1 can be defined by (9):

(13) $$\begin{array}{c} H^{\left(l\right)}=g\left(H^{\left(l-1\right)}W^{\left(l\right)}\right), \end{array}$$

whereas *W*^(1)^ ∈ ℝ^*d*×*N*^ and *W*^(*l*)^ ∈ ℝ^*N*×*N*^ imply the weighted matrices amongst the input-first and inter HL, correspondingly. Such variables (bias and weight) of hidden neurons were made arbitrarily in a suitable range and retained set from the trained stage. *g* signifies the nonlinear activation functions. Afterward, an input to resultant layers defined as follows:

(14) $$\begin{array}{c} D=\left[H^{\left(1\right)}H^{\left(2\right)}\ldots H^{\left(L-1\right)}H^{\left(L\right)}X\right]. \end{array}$$

This model structure corresponding to the RVFL network. Whereas input to output layers has nonlinear features in the stacked HL and novel features. Afterward, the resultant is defined as follows:

(15) $$\begin{array}{c} Y=D\beta_{d}. \end{array}$$

The resultant weighted *β*_*d*_ ∈ ℝ^(*NL*+*d*)×*K*^ (*K*: the count of classes) has been resolved. In (14) and (15), DRVFLN occurs a linear integration amongst the feature and resultant layer weighted matrix *β*_*d*_ which is the weight of the count of features from the HL containing the input layer.

## 3. Experimental Validation

The experimental validation of the OALOFS-MLC model is tested using two datasets namely German credit [20] and Australian credit [21] datasets. The former dataset includes 1,000 samples and 24 features. The latter dataset holds 690 instances with 14 features.

Table 1 offers the number of features selected by the OALOFS-MLC model on the applied datasets. The table values indicated that the OALOFS-MLC model that selected a total of 12 features for the German Credit dataset and 9 features for the Australian Credit dataset.

**Figure 3:**
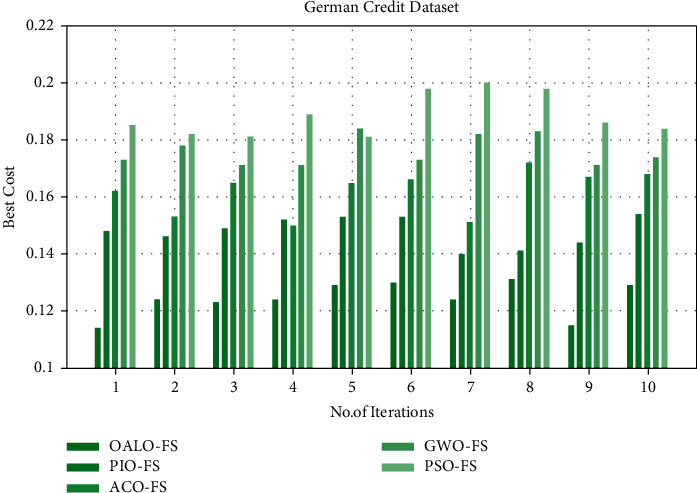
BC analysis of OALOFS-MLC technique under German credit dataset.

Table 2 and Figure 3 compare the best cost (BC) incurred by the OALOFS-MLC model on the test German Credit dataset. The experimental values implied that the OALOFS-MLC algorithm gained enhanced performance with the least BC values under all iterations. For instance, on iteration 1, the OALOFS-MLCapproach has obtained a lower BC of 0.114; the PIOFS system, ACOFS approach, GWOFS methodology, and PSOFSmodel have resulted in increased BC of 0.148, 0.162, 0.173, and 0.185, respectively.

In addition, on iteration 5, the OALOFS-MLC approach has obtained a lower BC of 0.129; the PIOFS system, ACOFS approach, GWOFS methodology, and PSOFS model have resulted in increased BC of 0.153, 0.165, 0.184, and 0.181, correspondingly. On iteration 10, the OALOFS-MLC algorithm has obtained a lesser BC of 0.129; the PIOFS system, ACOFS approach, GWOFS methodology, and PSOFS techniques have resulted in maximal BC of 0.154, 0.168, 0.174, and 0.184, correspondingly.

**Figure 4:**
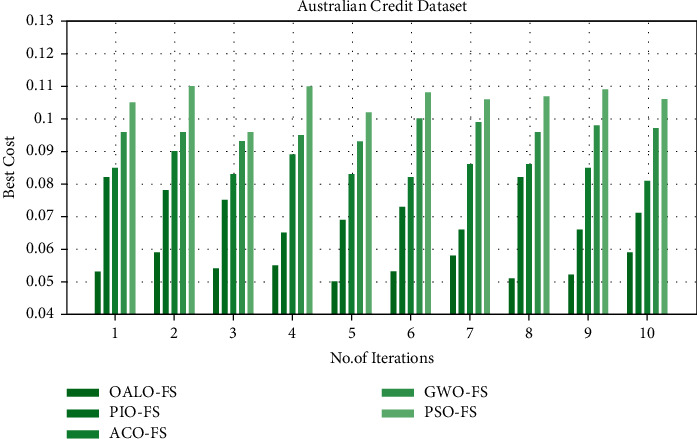
Analysis of OALOFS-MLC technique under Australian credit dataset.

Table 3 and Figure 4 relate the BC gained by the OALOFS-MLC method on the test German Credit dataset. The experimental values represented OALOFS-MLC system has improved performance with the least BC values under all iterations. For instance, on iteration 1, the OALOFS-MLC methodology has obtained a reduced BC of 0.053; the PIOFS system, ACOFS approach, GWOFS methodology, and PSOFS model have resulted in maximal BC of 0.082, 0.085, 0.096, and 0.105, respectively. Besides, on iteration 5, the OALOFS-MLC system has obtained a lower BC of 0.050; the PIOFS system, ACOFS approach, GWOFS methodology, and PSOFS model have resulted in increased BC of 0.069, 0.089, 0.093, and 0.102, respectively.

Likewise, on iteration 10, the OALOFS-MLC methodology has reduced BC of 0.059; the PIOFS system, ACOFS approach, GWOFS methodology, and PSOFS model have resulted in superior BC of 0.071, 0.081, 0.097, and 0.106, correspondingly.

Table 4 offers a detailed comparative examination of the FCP outcomes of the OALOFS-MLC model with recent models on the German Credit dataset [22].

**Figure 5:**
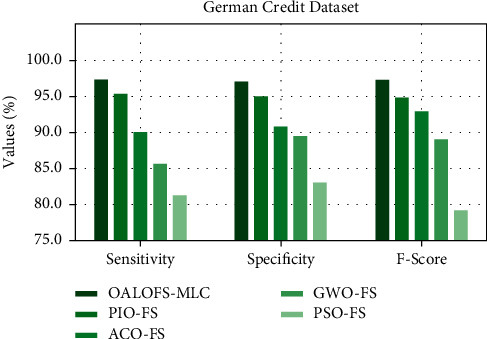
Sens_*y*_, Spec_*y*_, and *F*_−score_ analysis of OALOFS-MLC approach on German credit dataset.

Figure 5 provides a comparative study of the OALOFS-MLC model based on sens_*y*_, spec_*y*_, and *F*_−score_. The figure indicated that the OALOFS-MLC model reached maximum classification performance. Regarding sens_*y*_, the OALOFS-MLC model has achieved a higher sens_*y*_ of 97.36%; the PIOFS system, ACOFS approach, GWOFS methodology, and PSOFS model have obtained a lower sens_*y*_ of 95.43%, 90.12%, 85.73%, and 81.28%, respectively. Also, aboutspec_*y*_, the OALOFS-MLCapproach has gained a superior spec_*y*_ of 97.06%; the PIOFS system, ACOFS approach, GWOFS methodology, and PSOFS model have obtained a lower spec_*y*_ of 95.06%, 90.82%, 89.48%, and 83.02%, correspondingly. In terms of *F* − score, the OALOFS-MLC system has achieved a higher *F*_−score_ of 97.31%; the PIOFS system, ACOFS approach, GWOFS methodology, and PSOFS model have obtained a lower *F*_−score_ of 94.88%, 92.93%, 89.31%, and 79.17%, correspondingly.

**Figure 6:**
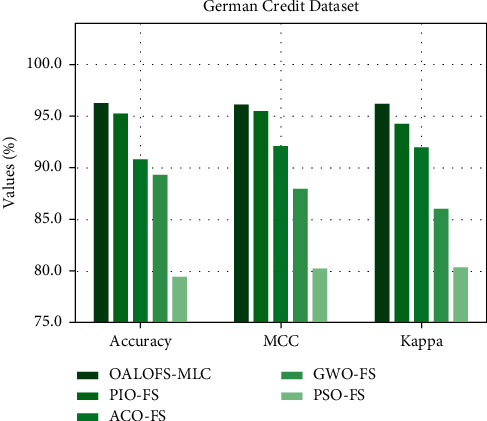
Accu_*y*_, MCC, and Kappa analysis of OALOFS-MLC approach on German credit dataset.

Figure 6 illustrates a comparison study of the OALOFS-MLC model with recent techniques in terms of accu_*y*_, MCC, and kappa. The figure represented that the OALOFS-MLC approach has obtained maximal classification performance. In terms of accu_*y*_, the OALOFS-MLC algorithm has achieved a superior accu_*y*_ of 98.75%, but the PIOFS system, ACOFS approach, GWOFS methodology, and PSOFS model have obtained minimal accu_*y*_ of 95.23%, 90.81%, 89.31%, and 79.42%, correspondingly. Moreover, concerning MCC, the OALOFS-MLC algorithm has achieved a higher MCC of 96.13% whereas the PIOFS system, ACOFS approach, GWOFS methodology, and PSOFS model have obtained a lower MCC of 95.47%, 92.12%, 87.97%, and 80.22%, correspondingly. In addition, in terms of kappa, the OALOFS-MLC system has achieved higher kappa of 96.19%, whereas the PIOFS system, ACOFS approach, GWOFS algorithm, and PSOFS methodologies have obtained lower kappa of 94.24%, 91.94%, 85.98%, and 80.36%, correspondingly.

**Figure 7:**
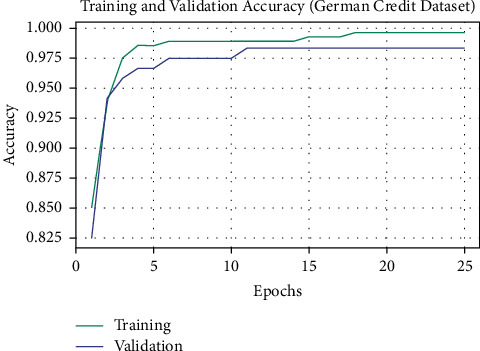
TA and VA analysis of OALOFS-MLC approach under German credit dataset.

The training accuracy (TA) and validation accuracy (VA) attained by the OALOFS-MLC approach on the German Credit dataset are demonstrated in Figure 7. The experimental outcome implied that the OALOFS-MLC system has gained maximum values of TA and VA. In specific, the VA seemed to be higher than TA.

**Figure 8:**
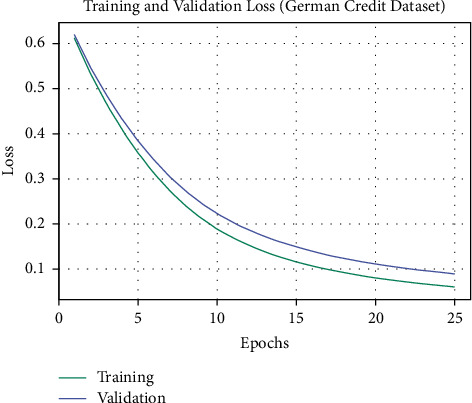
TL and VL analysis of OALOFS-MLC approach under German credit dataset.

The training loss (TL) and validation loss (VL) achieved by the OALOFS-MLC algorithm on the German Credit dataset are established in Figure 8. The experimental outcome inferred that the OALOFS-MLC methodology has been least values of TL and VL. In specific, the VL seemed to be lower than TL.

Table 5 offers a detailed comparative investigation of the FCP outcomes of the OALOFS-MLC algorithm with recent systems on the overall work.

**Figure 9:**
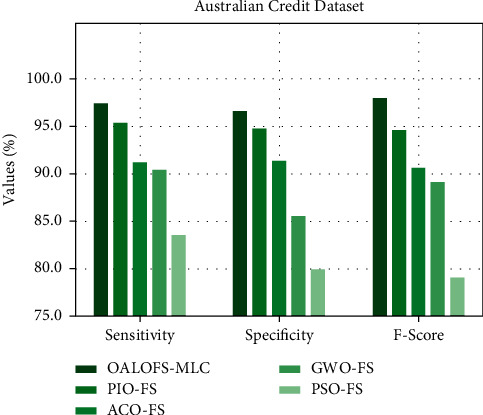
Sens_*y*_, Spec_*y*_, and *F*_−score_ analysis of OALOFS-MLC approach on Australian credit dataset.

Figure 9 provides at Table 5 comparative study of the OALOFS-MLC system with recent methodologies for sens_*y*_, spec_*y*_, and *F*_−score_. The figure indicated that the OALOFS-MLC model has reached higher classification performance. In terms of sens_*y*_, the OALOFS-MLC system has achieved a superior sens_*y*_ of 97.41%, whereas the PIOFS system, ACOFS approach, GWOFS methodology, and PSOFS model have obtained a lesser sens_*y*_ of 95.36%, 91.18%, 90.43%, and 83.51%, correspondingly. Also, for spec_*y*_, the OALOFS-MLC system has achieved a higher spec_*y*_ of 96.53%, whereas the PIOFS system, ACOFS approach, GWOFS methodology, and PSOFS model have obtained minimal spec_*y*_ of 94.71%, 91.28%, 85.52%, and 79.93%, correspondingly. Eventually, concerning *F* − score, the OALOFS-MLC methodology has achieved a higher *F*_−score_ of 97.92%, whereas the PIOFS system, ACOFS approach, GWOFS methodology, and PSOFS model have obtained decreased *F* − score of 94.61%, 90.65%, 89.07%, and 79.06%, correspondingly.

**Figure 10:**
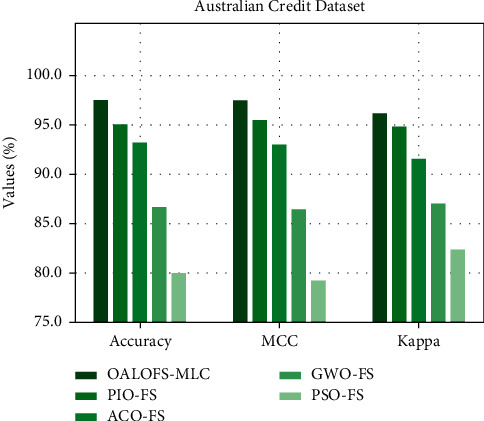
Accu_*y*_, MCC, and Kappa analysis of OALOFS-MLC approach on Australian credit dataset.

Figure 10 depicts a comparison study of the OALOFS-MLC model with recent methodologies in terms of accu_*y*_, MCC, and kappa. The figure represents that the OALOFS-MLC model has gained maximal classification performance. Concerning accu_*y*_, the OALOFS-MLC system has achieved a higher accu_*y*_ of 98.50%, whereas the PIOFS system, ACOFS approach, GWOFS methodology, and PSOFS model have obtained a lower accu_*y*_ of 95.06%, 93.18%, 86.65%, and 79.95%, correspondingly. Also, concerning MCC, the OALOFS-MLC approach has achieved a superior MCC of 97.53%, whereas the PIOFS system, ACOFS approach, GWOFS methodology, and PSOFS model have obtained lower MCC of 95.50%, 93.08%, 86.41%, and 79.19%, respectively. At last, concerning kappa, the OALOFS-MLC approach has achieved higher kappa of 96.22%, whereas the PIOFS system, ACOFS approach, GWOFS algorithm, and PSOFS methodology have obtained lower kappa of 94.87%, 91.64%, 87%, and 82.40%, correspondingly.

**Figure 11:**
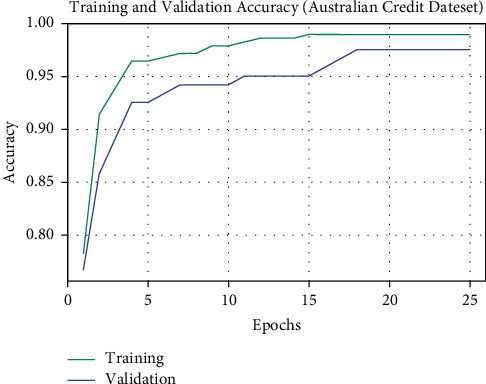
TA and VA analysis of OALOFS-MLC approach under Australian credit dataset.

The TA and VA obtained by the OALOFS-MLC model on the Australian Credit dataset are established in Figure 11. The experimental outcome outperformed that the OALOFS-MLC methodology has gained maximal values of TA and VA. In specific, the VA seemed that superior to TA.

**Figure 12:**
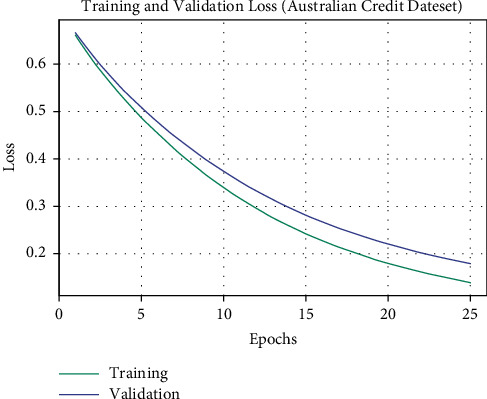
TL and VL analysis of OALOFS-MLC approach under Australian credit dataset.

The TL and VL attained by the OALOFS-MLC approach on the Australian Credit dataset are established in Figure 12. The experimental outcome signified that the OALOFS-MLC system has accomplished minimal values of TL and VL. In specific, the VL seemed to be lower than TL.

From the detailed results and discussion, it can be stated that the OALOFS-MLC model has shown an effectual outcome on FCP.

## 4. Conclusion

In this study, a novel OALOFS-MLC model was established for FCP in a big data environment. To handle the big data in the financial sector, the Hadoop MapReduce tool is employed. Besides, the presented OALOFS-MLC model designs a novel OALOFS algorithm for choosing an optimum subset of features which helps in attaining improved classification results. Furthermore, the DRVFLN model is exploited to perform the classification process. The experimental validation of the OALOFS-MLC approach was performed utilizing a benchmark dataset and the outcomes highlighted the supremacy of the OALOFS-MLC model over recent approaches. Thus, the presented OALOFS-MLC model can be exploited as an effectual tool for FCP in the big data environment. In the future, outlier detection and data clustering approaches can be applied to FCP.

## Figures and Tables

**Table 1:** Dataset details.

Dataset	Selected features
German credit	1, 3, 6, 7, 8, 9, 10, 12, 14, 18, 21, 22
Australian credit	1, 2, 4, 7, 8, 9, 10, 11, 13

**Table 2:** BC analysis of OALOFS-MLC technique with existing approaches on German credit dataset.

Best cost—German credit dataset					
No. Of iterations	OALO-FS	PIO-FS	ACO-FS	GWO-FS	PSO-FS
1	0.114	0.148	0.162	0.173	0.185
2	0.124	0.146	0.153	0.178	0.182
3	0.123	0.149	0.165	0.171	0.181
4	0.124	0.152	0.150	0.171	0.189
5	0.129	0.153	0.165	0.184	0.181
6	0.130	0.153	0.166	0.173	0.198
7	0.124	0.140	0.151	0.182	0.200
8	0.131	0.141	0.172	0.183	0.198
9	0.115	0.144	0.167	0.171	0.186
10	0.129	0.154	0.168	0.174	0.184

**Table 3:** BC analysis of OALOFS-MLC technique with existing approaches on Australian credit dataset.

Best cost—Australian credit dataset					
No. Of iterations	OALO-FS	PIO-FS	ACO-FS	GWO-FS	PSO-FS
1	0.053	0.082	0.085	0.096	0.105
2	0.059	0.078	0.090	0.096	0.110
3	0.054	0.075	0.083	0.093	0.096
4	0.055	0.065	0.089	0.095	0.110
5	0.050	0.069	0.083	0.093	0.102
6	0.053	0.073	0.082	0.100	0.108
7	0.058	0.066	0.086	0.099	0.106
8	0.051	0.082	0.086	0.096	0.107
9	0.052	0.066	0.085	0.098	0.109
10	0.059	0.071	0.081	0.097	0.106

**Table 4:** Comparative analysis of OALOFS-MLC algorithm with existing methodologies on German credit dataset.

German credit dataset						
Methods	Sensitivity	Specificity	Accuracy	F-score	MCC	Kappa
OALOFS-MLC	97.36	97.06	98.75	97.31	96.13	96.19
PIO-FS	95.43	95.06	95.23	94.88	95.47	94.24
ACOFS	90.12	90.82	90.81	92.93	92.12	91.94
GWOFS	85.73	89.48	89.31	89.08	87.97	85.98
PSO-FS	81.28	83.02	79.42	79.17	80.22	80.36

**Table 5:** Comparative analysis of overall work.

Overall performance measures						
Methods	Sensitivity	Specificity	Accuracy	F-score	MCC	Kappa
OALOFS-MLC	97.41	96.53	98.50	97.92	97.53	96.22
PIO-FS	95.36	94.71	95.06	94.61	95.50	94.87
ACOFS	91.18	91.28	93.18	90.65	93.08	91.64
GWOFS	90.43	85.52	86.65	89.07	86.41	87.00
PSO-FS	83.51	79.93	79.95	79.06	79.19	82.40

## Data Availability

All data are available in the article.

## References

[1] Uthayakumar J., Metawa N., Shankar K., Lakshmanaprabu S. K. (2020). Financial crisis prediction model using ant colony optimization. *International Journal of Information Management*.

[2] Donepudi P. K., Banu M. H., Khan W., Neogy T. K., Asadullah A. B. M., Ahmed A. A. A. (2020). Artificial intelligence and machine learning in treasury management: a systematic literature review. *International Journal of Management*.

[3] Vadlamudi S. (2020). The impacts of machine learning in financial crisis prediction. *Asian Business Review*.

[4] Samitas A., Kampouris E., Kenourgios D. (2020). Machine learning as an early warning system to predict financial crisis. *International Review of Financial Analysis*.

[5] Uthayakumar J., Metawa N., Shankar K., Lakshmanaprabu S. K. (2020). Intelligent hybrid model for financial crisis prediction using machine learning techniques. *Information Systems and E-Business Management*.

[6] Goodell J. W., Kumar S., Lim W. M., Pattnaik D. (2021). Artificial intelligence and machine learning in finance: identifying foundations, themes, and research clusters from bibliometric analysis. *Journal of Behavioral and Experimental Finance*.

[7] Alareeni B. (2019). A review of auditors’ GCOs, statistical prediction models and artificial intelligence technology. *International Journal of Business Ethics and Governance*.

[8] Bluwstein K., Buckmann M., Joseph A., Kapadia S., Simsek Ö. (2021). Credit Growth, the Yield Curve, and Financial Crisis Prediction:Evidence from a Machine Learning Approach.

[9] Kim S., Ku S., Chang W., Song J. W. (2020). Predicting the direction of US stock prices using effective transfer entropy and machine learning techniques. *IEEE Access*.

[10] Beutel J., List S., von Schweinitz G. (2019). Does machine learning help us predict banking crises?. *Journal of Financial Stability*.

[11] El-Kenawy E. S. M., Eid M. M., Saber M., Ibrahim A. (2020). MbGWO-SFS: modified binary grey wolf optimizer based on stochastic fractal search for feature selection. *IEEE Access*.

[12] Kolesnik J., Nadolski J. (2021). Optimization of the bank’s value in conditions of globalisation and permanent crisis. *European Research Studies Journal*.

[13] Bonivento L. (2021). Supply Chain Finance. *A New Approach to Enhance NWC Optimization*.

[14] Wang Y., Chen R., Lu Y. (2022). Dynamic Early Warning Method of Multi Classification Financial Crisis of Listed Companies Based on Particle Swarm Optimization. *Wireless Communications and Mobile Computing*.

[15] Latchoumi T. P., Swathi R., Vidyasri P., Balamurugan K. Develop new algorithm to improve safety on WMSN in health disease monitoring.

[16] Drenovak M., Ranković V., Urošević B., Jelic R. (2022). Mean-maximum drawdown optimization of buy-and-hold portfolios using a multi-objective evolutionary algorithm. *Finance Research Letters*.

[17] Ding R., Uryasev S. (2022). Drawdown beta and portfolio optimization. *Quantitative Finance*.

[18] Zhai Y., Shi P. (2022). The evolutionary characteristics, driving mechanism, and optimization path of China’s tourism support policies under COVID-19: a quantitative analysis based on policy texts. *Current Issues in Tourism*.

[19] Karnan B., Kuppusamy A., Latchoumi T. P. (2022). Multi-response Optimization of Turning Parameters for Cryogenically Treated and Tempered WC–Co Inserts. *Journal of The Institution of Engineers (India):Series D*.

[20] Bouchekourte M., El Hami N. (2022). Optimization of equity allocations of institutional investors: study of Moroccan case. *International Journal for Simulation and Multidisciplinary Design Optimization*.

[21] Pugazhendhi L. T., Kothandaraman R., Karnan B. (2022). Implementation of visual clustering strategy in self-organizing Map for wear studies samples printed using FDM. *Traitement du Signal*.

[22] Metawa N., Nguyen P. T., Le Hoang Thuy To Nguyen Q., Elhoseny M., Shankar K. (2021). Internet of Things enabled financial crisis prediction in enterprises using optimal feature subset selection-based classification model. *Big Data*.

